# Pharmacokinetics, Optimal Dosages, and Withdrawal Time of Florfenicol in Cobia ( *Rachycentron canadum* ) After Oral Administration via Medicated Feed

**DOI:** 10.1111/jvp.70049

**Published:** 2026-01-31

**Authors:** Jou‐An Shih, Tirawat Rairat, Yi‐Ping Lu, Chi‐Ming Wu, Niti Chuchird, Chi‐Chung Chou

**Affiliations:** ^1^ Department of Veterinary Medicine, College of Veterinary Medicine National Chung Hsing University Taichung Taiwan; ^2^ Department of Fishery Biology, Faculty of Fisheries Kasetsart University Bangkok Thailand; ^3^ Biology Division, Veterinary Research Institute Ministry of Agriculture New Taipei City Taiwan; ^4^ Department and Graduate Institute of Pharmacology National Defense Medical Center Taipei Taiwan

**Keywords:** antimicrobials, florfenicol, marine aquaculture, veterinary drug

## Abstract

Antimicrobial drugs are commonly used for the treatment of bacterial diseases in cobia ( *Rachycentron canadum* ), but information regarding the rationale of their therapeutic use, such as pharmacokinetics (PK), optimal dosages, and withdrawal time (WDT) in this species is very rare. The present study evaluated the PK characteristics of florfenicol (FF) in cobia at 25°C after a single oral administration of 10 mg/kg via medicated feed. FF in the serum was determined by an HPLC method and the PK parameters were analyzed by a one‐compartmental model. In tissue depletion and drug WDT study, cobia were fed with FF medicated feed at 10 mg/kg once daily for 5 days. The WDT was determined by linear regression analysis using the sum of FF and its metabolite florfenicol amine as the marker residue. The results revealed that FF has desirable PK characteristics in cobia, including a high peak serum concentration (*C*_max_, 9.08 μg/mL), a large area under the serum concentration‐time curve (AUC, 182.68 h·μg/mL), and a moderately long elimination half‐life (*t*_1/2K_, 10.22 h). The optimal dosage for a minimum inhibitory concentration (MIC) of 2 μg/mL at 25°C was 6.23 mg/kg/day. The WDT was calculated to be 5 days based on muscle/skin and 6 days based on serum depletion. Since serum sampling can reduce animal use, it warrants further investigation despite not being an official target tissue for WDT. Our findings indicated that FF is a good choice for treating bacterial diseases in cobia due to its favorable PK profile and short WDT.

## 1 Introduction

Cobia ( *Rachycentron canadum* ) is the only known species in the family Rachycentridae. While it was formerly classified within the order Perciformes (Carangoid lineage) (Nelson 2006), it is now placed in the order Carangiformes (Nelson et al. 2016). This fish can be found in tropical and subtropical waters worldwide except for the central and eastern Pacific. It is a pelagic fish, but it is also found in coastal and continental shelf waters. It is an active predator that primarily feeds on crustaceans, but its diet also includes squids and fish (Kaiser and Holt 2007; Benetti et al. 2021; NOAA Fisheries 2025). Cobia is an important aquaculture species in East Asia due to its high economic value. The production systems include larval rearing ponds, outdoor ponds or nearshore cages (the nursery phase), and grow‐out cages (the grow‐out phase) until the fish reach a market size of 6–10 kg (Liao et al. 2004; Kaiser and Holt 2007). In 2023, global aquaculture production of cobia was 42,863 t, with China being the largest producer (33,210 t), followed by Vietnam (6410 t), Taiwan (2230 t), and Panama (approximately 1000 t) (FAO 2025).

Cobia is susceptible to a variety of infectious diseases, including those caused by pathogenic bacteria such as *Vibrio harveyi* , *V. alginolyticus* , *V. vulnificus* , *V. parahaemolyticus* , *Photobacterium damselae* subsp. *damselae*, and *P. damselae* subsp. *piscicida* (Rajan et al. 2001; Lopez et al. 2002; Liu et al. 2004; Rameshkumar et al. 2017; Sulumane Ramachandra et al. 2021). When a disease outbreak occurs, farmers may use antibiotics to prevent fish deaths. Unfortunately, our knowledge on antibiotic use in cobia is very limited. Rational drug use, which maximizes drug efficacy while minimizing undesirable effects, requires information on drug pharmacokinetics (PK), optimal dosages, and appropriate withdrawal time (WDT). To date, only partial information on the WDT of oxolinic acid in cobia has been reported by one study (Chen et al. 2019). Following medicated feed administration of oxolinic acid at 30 and 60 mg/kg/day for 5 days, a WDT of 15 days was suggested based on a 10‐day depletion time (in the liver) plus a 5‐day safety span. However, since the study did not consider the muscle/skin as a target tissue, and did not include linear regression analysis—a standard method for WDT determination—the reported WDT information is incomplete. To the best of the authors' knowledge, no information is available regarding the PK and optimal dosages of any antimicrobial drug in cobia.

Florfenicol (FF) is one of the most common veterinary antibacterial drugs approved for use in food fish in many countries including the USA (U.S. FDA 2025), Japan (MAFF 2025), China (MARA, NFTEC, and CAFS 2024), and Taiwan (COA 2025). Information on PK characteristics of FF is available for many fish species such as Nile tilapia ( *Oreochromis niloticus* ) (Rairat, Hsieh, Thongpiam, Sung, and Chou 2019; Rairat et al. 2022; Bardhan et al. 2024), Asian seabass ( *Lates calcarifer* ) (Rairat, Kumphaphat, et al. 2023; Rairat, Hsieh, et al. 2023), crucian carp ( *Carassius auratus* ) (Yang et al. 2019), European seabass ( *Dicentrarchus labrax* ) (Kogiannou et al. 2021), snubnose pompano ( *Trachinotus blochii* ) (Sumithra et al. 2023), and striped catfish (*Pangasianodon hypophthalmus*) (Pham et al. 2023). The PK data are necessary for determination of the optimal dosing regimen, which depends on fish species, water temperature, and the minimum inhibitory concentration (MIC) of the infecting bacteria (Rairat, Hsieh, Thongpiam, and Chou 2019; Rairat et al. 2022; Rairat, Kumphaphat, et al. 2023; Rairat, Hsieh, et al. 2023). The optimal doses of FF have been recently reported in Nile tilapia and Asian seabass. For example, at a rearing temperature of about 25°C and an MIC of 2 μg/mL, the determined optimal dosages of FF were 2.98–4.45 mg/kg/day in Nile tilapia (Rairat, Hsieh, Thongpiam, and Chou 2019; Rairat et al. 2022), and 10.9–13.38 mg/kg/day in Asian seabass (Rairat, Kumphaphat, et al. 2023; Rairat, Hsieh, et al. 2023), respectively. The general recommended dosage of FF in fish by the U.S. FDA (2025) is 10–15 mg/kg/day for 10 days. The maximum residue limit (MRL) of FF in fish is 1 μg/g, targeting muscle tissue with the natural portion of skin, as specified by the U.S. FDA (2025), the EC (2025), and the Taiwan FDA (2023). However, the designated marker residue varies across regulatory authorities. The parent drug FF is considered the marker residue in Taiwan (Taiwan FDA 2023); FFA is used in the USA (CFR 2025); the sum of FF and all its metabolites expressed as FFA is applied in the European Union (EC 2025); and FF + FFA is used in China (MARA 2019). In Taiwan, guidelines for approved drug use in aquaculture, including recommended dosages and WDT, differ by fish taxonomic order (COA 2025). Regarding FF, although a standard dosing regimen of 10 mg/kg/day for 3–5 days is applied for all fish, the WDT differs significantly among different taxonomic orders: 5 days in Cypriniformes and Siluriformes, 7 days in Anguilliformes, 14 days in Salmoniformes, 15 days in Perciformes, Acipenseriformes, and Gonorynchiformes (COA 2025). Notably, Taiwan's Council of Agriculture (Executive Yuan) still classifies cobia under the order Perciformes; therefore, the suggested WDT for FF in cobia is 15 days. This aligns with the 15‐day WDT established by the U.S. FDA (2025).

The objectives of the current study were to determine the PK characteristics of FF and its optimal dosing regimen in cobia reared at 25°C following a single oral dose of 10 mg/kg FF via medicated feed. Tissue depletion and the appropriate WDT of FF were also studied. The outcomes of this study are important for the prudent use of FF in controlling bacterial diseases in cobia aquaculture.

## 2 Materials and Methods

### 2.1 Chemicals

Reference standards of florfenicol (FF) and florfenicol amine (FFA), along with sodium dodecyl sulfate and ammonium hydroxide (NH_4_ OH), were obtained from Sigma‐Aldrich (St. Louis, MO, USA). HPLC‐grade acetonitrile was sourced from Avantor Performance Materials (Center Valley, PA, USA). Triethylamine was acquired from Alfa Aesar, Thermo Fisher Scientific (Heysham, Lancashire, UK). Sodium dihydrogen phosphate anhydrous (NaH_2_ PO_4_) was purchased from Panreac Química SLU (Barcelona, Spain), while 85% phosphoric acid (H_3_ PO_4_) was provided by Scharlau (Barcelona, Spain).

### 2.2 Experimental Fish

Fifty‐eight healthy cobia with an average weight of 500 ± 100 g were obtained from a commercial aquaculture facility in Pingtung County, Taiwan. The fish were housed in an indoor system at the Aquatic Animal Medicine Laboratory of the Veterinary Research Institute, under the Council of Agriculture, Taiwan. The fish were reared in seawater (35 ppt) maintained at 25°C. Water parameters, including temperature, pH (7.5–8.0), and dissolved oxygen (≥ 5.0 mg/L), were regularly monitored using a Lutron WA‐2017SD portable meter (Lutron Electronics, Coopersburg, PA, USA). For the PK study, 8 fish were kept individually in 120‐L tanks. For the tissue depletion study, 50 fish were used, with three fish allocated to each 240‐L tank. The fish were acclimated for 3 days prior to dosing and were offered feed at 0.12% of body weight to confirm that all individuals were actively feeding. The animal study was approved by the Institutional Animal Care and Use Committee of National Chung Hsing University (IACUC Approval No: 112‐113).

### 2.3 Drug Administration and Blood Collection

Commercial feed pellets (TRIFULL, Ho‐Yi Brand, Chuan Hsing Feed Co., Taiwan) used in this trial had the following approximate composition: 43% crude protein, 3% fat, 16% ash, 3% fiber, and ≤ 20% moisture. In this study, medicated feed was prepared individually for each fish. A base amount of feed equivalent to 0.12% of the fish's body weight was used, with each pellet weighing approximately 0.3 g. FF stock solution was prepared at a concentration of 100 mg/mL. Based on the target dosage of 10 mg FF per kg of body weight (BW), the required volume of FF solution was calculated for each fish and applied directly onto the feed pellets. Typically, two pellets were sufficient to absorb the entire calculated volume. After the FF solution was added, the pellets were allowed to fully absorb the liquid. During administration, medicated pellets were offered one by one to each fish, and ingestion was visually confirmed. Only fish that swallowed every medicated pellet without regurgitation were considered successfully dosed. For the PK study, blood samples (approximately 0.4–0.5 mL) were drawn from the caudal vessel using the lateral approach (Grant 2015) at the following time intervals: 0.25, 0.5, 1, 2, 4, 8, 12, 24, 36, 48, 60, and 72 h post‐treatment. This sampling strategy is generally regarded as acceptable in fish PK studies and is not expected to overtly affect the physiology of fish of this size. The cumulative blood volume collected (4.8–6 mL across 12 samplings, corresponding to ~19%–24% of total blood volume by 72 h) remains within physiologically safe limits as described by Grant (2015). Samples were left to coagulate at room temperature, centrifuged (2000 *g*, 10 min), and the separated serum was stored at −20°C for later analysis.

### 2.4 Sample Processing and HPLC Analysis

All samples were frozen at −80°C immediately after collection and analyzed within 2 weeks. FF levels in serum and muscle/skin were analyzed using an HPLC‐FLD method based on previously reported procedures (Rairat et al. 2022). Two extractions were performed on 200 μL of serum or 1 g homogenized muscle/skin samples using 600 μL or 3 mL acetonitrile: ammonium hydroxide (98:2, v/v), respectively, followed by centrifugation (2000 *g*, 10 min). Supernatants were evaporated at 45°C using a sample concentrator (SP Genevac miVac Duo Concentrator, Suffolk, England), reconstituted with 200 μL or 1 mL of the mobile phase, and filtered through a 0.2 μm nylon syringe filter. The mobile phase consisted of acetonitrile and a phosphate buffer (10 mM NaH_2_PO_4_, 5 mM sodium dodecyl sulfate, 0.01% triethylamine, adjusted to pH 4.8 with 85% phosphoric acid) in a 35:65 v/v ratio. The HPLC system (Agilent 1260 Infinity II) included a fluorescence detector (G7121A), autosampler (G7129A), and C‐18 analytical column (150 × 4.6 mm, 5 μm particle size, Apollo). The flow rate was set at 1 mL/min, with detection wavelengths at 233 nm (excitation) and 284 nm (emission). Each sample injection volume was 50 μL.

To construct calibration curves for quantifying FF and FFA across various matrices, linearity was first confirmed using standard solutions in the range of 0.05–25 ppm (*n* = 6). For matrix‐matched validation, FF and FFA were spiked into cobia serum (0.1–25 ppm) or skin‐on muscle (0.1–5 ppm). All samples were extracted as described above and analyzed using the described HPLC procedure, with a 1/*x* ^2^ weighting factor applied to improve the accuracy at lower concentrations. The limits of detection (LOD) and quantification (LOQ) were calculated as 3.3 and 10 σ/S, respectively, where σ refers to the standard deviation of the y‐intercepts obtained from calibration curves and S is the slope of the calibration curve. In serum, the calibration curves for FF and FFA were linear between 0.1 and 25 ppm, with *r* ^2^ values of 0.9987 and 0.9986. Their LOD and LOQ were 10 and 40 ppb for both analytes. Precision did not exceed 7%, and extraction recoveries ranged from 95% to 102% for FF and 81%–90% for FFA. In skin‐on muscle, linearity was verified from 0.1 to 5 ppm, with *r* ^2^ values of 0.9964 (FF) and 0.9967 (FFA). FF exhibited an LOD of 30 ppb and LOQ of 80 ppb; FFA showed an LOD of 20 ppb and an LOQ of 70 ppb. Precision remained below 6% for FF and 4% for FFA, and recoveries were 86%–94% for FF and 80%–85% for FFA (Table S1 and Figure S1).

### 2.5 Pharmacokinetic Analysis and Determination of Optimal Dosing Regimens

PK data were modeled using a one‐compartment framework and analyzed with PKSolver 2.0 (China Pharmaceutical University, Nanjing, China) under a 1/C weighting scheme (Zhang et al. 2010). The calculated parameters included: absorption rate constant (Ka), absorption half‐life (*t*_1/2Ka_), elimination rate constant (K), elimination half‐life (*t*_1/2K_), maximum serum concentration (*C*_max_), time to *C*_max_ (*T*_max_), area under the curve (AUC), volume of distribution relative to bioavailability (Vd/F), clearance relative to bioavailability (CL/F), and mean residence time (MRT).

To determine appropriate FF dosing at a defined MIC, a pharmacokinetic‐pharmacodynamic (PK/PD) approach was used, following previously established methodology (Rairat, Hsieh, Thongpiam, and Chou 2019). For time‐dependent bacteriostatic drugs without a prolonged post‐antibiotic effect (including FF), the most appropriate PK/PD index is *T* > MIC (the duration for which the drug concentration exceeds the MIC) (AliAbadi and Lees 2000; Martinez et al. 2012). PK parameters (Ka, K, and Vd/F) obtained from the current study were applied in the multiple‐dose equation for extravascular administration. The target MIC values were set at 1, 2, 3, and 4 μg/mL to represent a range of potential antimicrobial susceptibility levels.

### 2.6 Tissue Depletion Study

The fish were fed with the medicated feed for 5 days at a dosage of 10 mg/kg BW/day (U.S. FDA 2025). Tissue samples were collected on days 1, 3, 5, 8, and 11 post‐treatment from 10 fish per time point. Fish were euthanized via rapid decapitation and brain pithing (AVMA 2020). The muscle/skin samples were collected from the dorsal white muscle located lateral to the lateral line. The trunk of each fish was divided into anterior, middle, and posterior sections. From each section, a piece of skin‐on fillet muscle tissue measuring approximately 2 cm in length and 2 cm in width was excised at a depth extending from the epidermis to the surface of the bones, yielding a total sample weight of approximately 10–15 g. All collected pieces were then finely minced, and 1 g of the material was transferred into a 5‐mL tube for homogenization. The samples were subsequently stored at −80°C for analysis.

### 2.7 Determination of Withdrawal Time (WDT)

The WDT was calculated based on the combined concentrations of FF and FFA (marker residues) in muscle/skin. Linear regression was performed using WT 1.4 software, following the guidance of EMA (2018). WDT corresponds to the time when the upper 95% one‐sided tolerance limit, with 95% confidence, falls below the MRL of 1 μg/g. Concentrations below the LOQ were substituted with a value equal to one‐half of the LOQ (LOQ/2) for subsequent calculations. Degree‐days were calculated by multiplying WDT by water temperature. The depletion half‐life (*t*_1/2_) of FF + FFA was calculated by PKSolver 2.0.

## 3 Results

The PK characteristics of FF in the serum of cobia following a single oral dose of 10 mg/kg FF via medicated feed were best described by a one‐compartment model (Table 1). The model selection was based on visual inspection of the concentration–time profiles, comparison of Akaike's information criterion (AIC) values, and the precision of parameter estimates as reflected by their coefficients of variation (%CV) (Yamaoka et al. 1978; Riviere 1997; Gabrielsson and Weiner 1999). Overall, the 1‐compartment model yielded slightly lower AIC values than the 2‐compartment model (mean AIC: 3.66 vs. 5.11). More importantly, the %CV values of the estimated PK parameters were markedly lower in the 1‐compartment model (all ≤ 28%), whereas several parameters in the 2‐compartment model showed %CV values exceeding 100%. Given that the 1‐compartment model adequately captured the observed data and provided more precise parameter estimates, it was selected for the PK analysis.

**TABLE 1:** Pharmacokinetic parameters (mean ± SD) of 10 mg/kg florfenicol in cobia following medicated feed administration at 25°C (*n* = 8).

PK parameters	25°C
Ka (1/h)	0.54 ± 0.13
*t*_1/2Ka_ (h)	1.28 ± 0.37
K (1/h)	0.068 ± 0.008
*t*_1/2K_ (h)	10.22 ± 1.34
*C*_max_ (μg/mL)	9.08 ± 2.53
*T*_max_ (h)	4.55 ± 0.93
AUC (h·μg/mL)	182.68 ± 49.48
Vd/F (L/kg)	0.86 ± 0.21
CL/F (L/kg/h)	0.058 ± 0.015
MRT (h)	16.90 ± 2.30

*Note:* The PK parameters were determined by a 1‐compartmental model.

Abbreviations: AUC, area under the serum concentration‐time curve; CL/F, clearance relative to bioavailability; *C*_max_, maximum serum concentration; K, elimination rate constant; Ka, absorption rate constant; MRT, mean residence time; *t*_1/2K_, elimination half‐life; *t*_1/2Ka_, absorption half‐life; *T*_max_, time to reach *C*_max_; Vd/F, volume of distribution relative to bioavailability.

**FIGURE 1:**
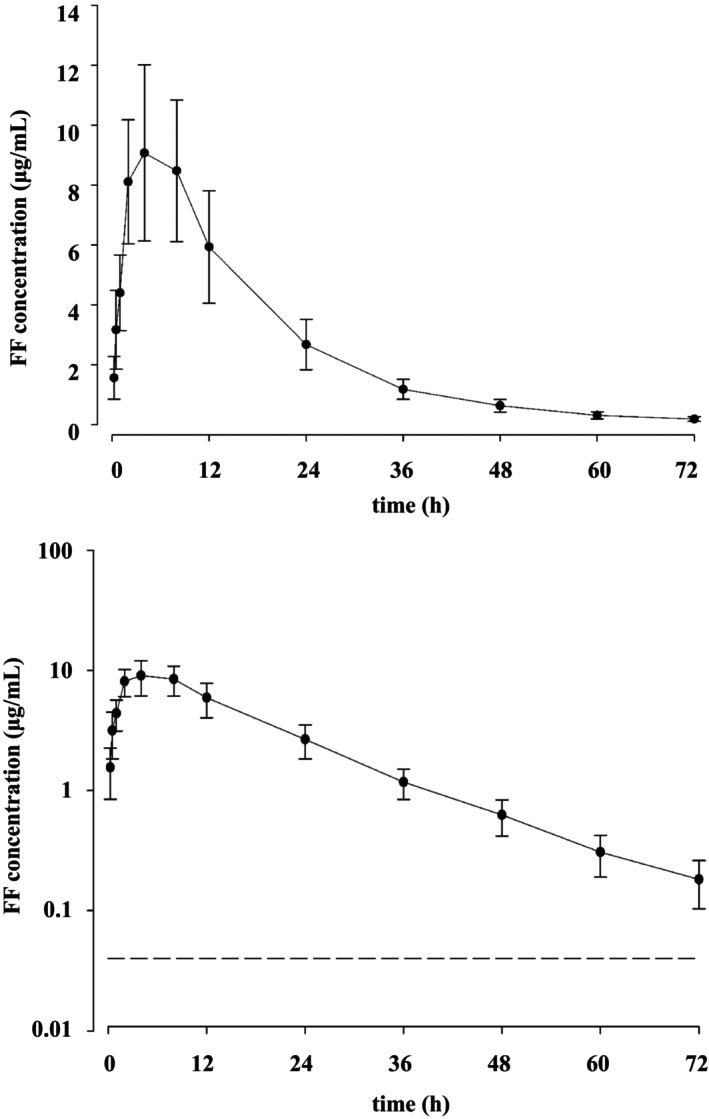
Linear (above) and semi‐logarithmic plots (below) of serum concentration‐time profile (mean ± SD) of 10 mg/kg florfenicol following medicated feed administration at 25°C (*n* = 8). The LOQ is indicated by a horizontal dotted line on the semi‐logarithmic plot.

The serum concentration‐time profiles of FF are presented in Figure 1 and Figure S2. FF reached a *C*_max_ of 9.08 μg/mL at a *T*_max_ of 4.55 h. The *t*_1/2Ka_ and *t*_1/2K_ were 1.28 and 10.22 h, respectively. The AUC was 182.68 h·μg/mL, the Vd/F was 0.86 L/kg, and the CL/F was 0.058 L/kg/h. The determined optimal dosages for MICs of 1, 2, 3, and 4 μg/mL were 3.11, 6.23, 9.34, and 12.46 mg/kg/day, respectively (Table 2).

**TABLE 2:** Determined optimal dosing regimens (mg/kg/day) of florfenicol in cobia (*n* = 8) at 25°C and different MIC values.

MIC value	Optimal dosing regimens (mg/kg/day)
MIC = 1 μg/mL	3.11 ± 1.09 (1.89–5.09)
MIC = 2 μg/mL	6.23 ± 2.18 (3.79–10.18)
MIC = 3 μg/mL	9.34 ± 3.27 (5.68–15.27)
MIC = 4 μg/mL	12.46 ± 4.36 (7.57–20.36)

*Note:* The optimal dosing regimens were presented as mean ± SD. The values in parentheses indicated the minimum and maximum range of the determined dosage.

**FIGURE 2:**
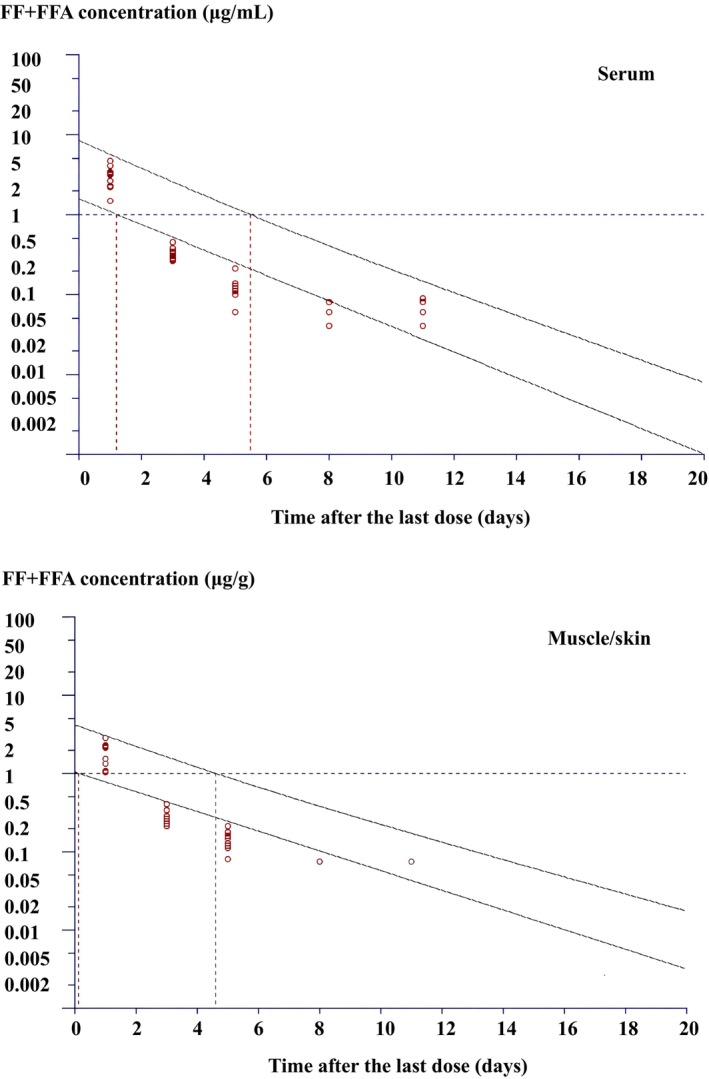
Tissue residue depletion of florfenicol (FF) plus florfenicol amine (FFA) in the serum (above) and muscle/skin (below) of cobia following multiple oral administrations of 10 mg/kg FF once daily for 5 days at 25°C (*n* = 10). The lower line represents the linear regression line, while the upper line represents its 95% upper tolerance limits with 95% confidence.

In the drug depletion study, the FF and FFA concentrations in the serum on Day 1 of the drug withdrawal (i.e., 24 h after the last medication) were 2.62 and 0.42 μg/mL, respectively (Table 3). At Day 5, they were depleted to 0.04 and 0.08 μg/mL, respectively. FFA in the serum was below the LOQ on Day 8. In the muscle/skin, FF and FFA on Day 1 were 1.43 and 0.47 μg/g, respectively. However, FF in the muscle/skin depleted below the LOQ since Day 3. Thus, its *t*_1/2_ could not be calculated. The muscle/skin FFA on Day 3 and 5 was 0.23 and 0.10 μg/g, respectively. The *t*_1/2_ of FFA in the muscle/skin was 43.65 h. The sum of FF + FFA, the marker residue, in the muscle/skin on Day 1 (1.89 μg/g) was still above the MRL of 1 μg/g. However, FF + FFA concentrations were lower than the MRL from Day 3 onward. The WDT was determined as 5 days (or 125°C‐days) (Figure 2). The depletion half‐life of FF + FFA in the muscle/skin was 17.27 h. Note that when WDT was calculated based on the serum concentration instead of the muscle/skin, it was determined to be 6 days (or 150°C‐days) (Figure 2).

**TABLE 3:** Florfenicol concentration in the serum (μg/mL) and muscle/skin (μg/g) of cobia following multiple oral administration of 10 mg/kg florfenicol once daily for 5 days at 25°C (*n* = 10) and their half‐lives (*t*_1/2_).

Time after the last dose	Serum				Muscle/skin			
	FF	FFA	FFA/FF	FF + FFA	FF	FFA	FFA/FF	FF + FFA
Day 1	2.62 ± 0.92	0.42 ± 0.05	0.18	3.04 ± 0.95	1.43 ± 0.57	0.47 ± 0.15	0.38	1.89 ± 0.61
Day 3	0.11 ± 0.03	0.21 ± 0.02	1.93	0.32 ± 0.06	0.06 ± 0.01^a^	0.23 ± 0.05	NA	0.29 ± 0.06
Day 5	0.04 ± 0.00	0.08 ± 0.04	1.78	0.12 ± 0.04	0.03 ± 0.00^a^	0.10 ± 0.04	NA	0.13 ± 0.04
Day 8	0.04 ± 0.01	0.03 ± 0.01^a^	NA	0.07 ± 0.01	0.03 ± 0.00^a^	0.03 ± 0.01^a^	NA	0.06 ± 0.01
Day 11	0.05 ± 0.01	0.02 ± 0.00^a^	NA	0.06 ± 0.01	0.03 ± 0.00^a^	0.02 ± 0.00^a^	NA	0.05 ± 0.00
*t*_1/2_ (h)	16.31	39.96	NA	20.77	NA	43.65	NA	17.27

Abbreviation: NA, not applicable.

^a^ Concentration below the LOQ (limit of quantification).

## 4 Discussion

Although cobia is a highly valuable farmed marine fish in East Asia and bacterial disease outbreaks are a serious problem, research on antibiotic use in cobia is lacking. This study is the first to comprehensively investigate the PK, optimal dosing regimen, and WDT of an antibacterial drug in cobia, providing crucial information for rational drug use. Our findings provide evidence supporting FF as an effective therapeutic agent for bacterial disease treatment in cobia from a pharmacokinetic point of view. Following oral administration of 10 mg/kg FF through medicated feed treatment, the serum concentration reached a relatively high *C*_max_ and large AUC. The dose‐normalized *C*_max_ (0.91 μg/mL) and dose‐normalized AUC (18.27 h·μg/mL) of cobia were greater than those of many fish species. For comparison, lower *C*_max_/dose and AUC/dose were observed in other fish reared at similar water temperatures (about 24°C–25°C) and treated using the same method (i.e., medicated feed); the reported *C*_max_/dose range from 0.052 μg/mL in three‐spot gourami ( *Trichogaster trichopterus* ) to 0.57 μg/mL in Asian seabass, and the AUC/dose range from 0.55 h·μg/mL in three‐spot gourami to 9.76 h·μg/mL in Asian seabass (Yanong et al. 2005; Kogiannou et al. 2021; Rairat, Kumphaphat, et al. 2023).

The *t*_1/2K_ of FF in cobia (10.22 h) was comparable to that of many fish species. In general, the *t*_1/2K_ of FF in fish reared at 24°C–25°C is usually in a range of 9–15 h. This pattern appears consistent across different fish species such as Nile tilapia (Rairat, Hsieh, Thongpiam, Sung, and Chou 2019; Rairat et al. 2022), Asian seabass (Rairat, Kumphaphat, et al. 2023; Rairat, Hsieh, et al. 2023), crucian carp ( *Carassius auratus* ) (Yang et al. 2019), European seabass ( *Dicentrarchus labrax* ) (Kogiannou et al. 2021), channel catfish ( *Ictalurus punctatus* ) (Gaunt et al. 2012), yellow catfish (*Tachysurus fulvidraco*) (Yang et al. 2013), Asian swamp eel ( *Monopterus albus* ) (Xie et al. 2013), and Japanese eel ( *Anguilla japonica* ) (Lin et al. 2015), with a few exceptions (Yanong et al. 2005; Wang et al. 2015). Given the potential dissimilarity in experimental conditions across these studies and the differences in the fish anatomy, physiology, ecology, and taxonomic position, the relatively similar half‐lives of FF in diverse fish are surprising. This consistency strongly implies that FF may undergo a similar elimination process across multiple fish species, with hepatic metabolism presumed to be the major route of drug elimination (Rairat et al. 2020).

The high serum concentration, large AUC, and moderately long *t*_1/2K_ of FF in cobia are favorable PK properties for effective antibiotic therapy. These properties of FF are generally consistent with other phylogenetically related fish, such as Nile tilapia (Rairat, Hsieh, Thongpiam, Sung, and Chou 2019; Rairat et al. 2022), Asian seabass (Rairat, Kumphaphat, et al. 2023; Rairat, Hsieh, et al. 2023), and European seabass (Kogiannou et al. 2021). It is worth noting that all of these species are currently classified, or were formerly classified, within the same taxonomic order, Perciformes (Nelson 2006; Nelson et al. 2016). This observation appears to support the practice of grouping fish by taxonomic order, as adopted by Taiwan's Council of Agriculture (Executive Yuan) (COA 2025). However, it should be interpreted with caution as this is not always the case. For instance, a PK study in snubnose pompano (order Carangiformes)—a species phylogenetically closest to cobia for which FF data are available—revealed a markedly different PK profile. Following a single oral medicated feed treatment at 15 mg/kg in snubnose pompano at 28.8°C, the *C*_max_ (0.80 μg/mL) and AUC (12.46 h·μg/mL) were considerably lower than in cobia and the aforementioned species.

The FF elimination from the muscle/skin of cobia was very rapid and fell below the LOQ from Day 3 onward. The FFA elimination was slower, a pattern consistent with general observation in many fish (Xie et al. 2013; Feng et al. 2016; Yang et al. 2020; Rairat et al. 2022; Rairat, Kumphaphat, et al. 2023; Rairat, Hsieh, et al. 2023). At 24 h of the drug withdrawal, the level of marker residue FF + FFA in the muscle/skin of cobia was 1.89 μg/g, which was lower than that of Asian seabass (2.00–3.03 μg/g) (Rairat, Kumphaphat, et al. 2023; Rairat, Hsieh, et al. 2023) and Nile tilapia (9.31 μg/g) (Rairat et al. 2022). The depletion *t*_1/2_ of FF + FFA in the muscle/skin of cobia (17.27 h) was also shorter than that of Asian seabass and Nile tilapia (41.1–68.33 h) (Rairat et al. 2022; Rairat, Kumphaphat, et al. 2023; Rairat, Hsieh, et al. 2023). The low concentration of FF + FFA combined with rapid elimination (short *t*_1/2_) results in a short WDT of FF in cobia (only 5 days). By comparison, the WDT of FF was 6–8 days in Asian seabass (Rairat, Kumphaphat, et al. 2023; Rairat, Hsieh, et al. 2023) and 10 days in Nile tilapia (Rairat, Kumphaphat, et al. 2023; Rairat, Hsieh, et al. 2023). Only a few species including crucian carp and European seabass have a shorter WDT (2–3 days) (Yang et al. 2020; Kogiannou et al. 2021) or 4 days in Pacu ( *Piaractus mesopotamicus* ) (Marques et al. 2018). Note that the marker residue of FF differs among regulatory authorities. We consider the use of FF or FFA alone to be overly permissive, whereas the EU approach (EC 2025), although comprehensive, requires a relatively complex conversion procedure. Given that other FF metabolites contribute minimally, FF + FFA represents the most practical and scientifically sound option (Rairat et al. 2022). Although fish serum is not typically considered a target tissue for human consumption and is therefore generally excluded from WDT determinations, it may be used as a surrogate for muscle/skin residues. In our study, the WDT determination based on the serum concentration was 6 days, very close to that determined from the muscle/skin (5 days). Using serum instead of muscle/skin potentially offers a significant advantage as blood can be collected repeatedly from the same individual fish, reducing the total number of animals used and avoiding the need to sacrifice them. This contrasts with the collection of muscle/skin, which requires sacrificing the fish. However, it should be noted that serum is not a typical target tissue for WDT estimation by regulators, and future studies are required to evaluate the feasibility of this approach. It is worth noting that the mean concentrations in WDT serum at the later time points (Day 8 and Day 11) showed a plateau or a slight elevation. This phenomenon may be attributed to the slow release of drug residues from tissues back into the bloodstream. It would be worthwhile to include an additional sampling point (e.g., extending the study by 1 day) in future studies to confirm the actual declining trend of the concentration. Considering Taiwan's regulation (COA 2025), a standard dose of FF at 10 mg/kg/day appears suitable for treating cobia infected with bacterial pathogens, provided their MIC is no greater than 2–3 μg/mL. For bacteria with an MIC of 4 μg/mL, a higher dose (i.e., 15 mg/kg/day) is required. Nevertheless, the recommended WDT of 15 days is unnecessarily long for cobia and warrants reconsideration.

To the best of our knowledge, although the current study represents the first investigation of the PK of FF in cobia following oral administration, some limitations are worth noting. First, the oral bioavailability of FF, as well as its absolute values of Vd and CL (rather than Vd/F and CL/F), remain unknown. Intravenous administration of FF is the only method to accurately assess the absolute bioavailability, and this approach should be considered in future studies. In addition, an in vitro antimicrobial susceptibility test of cobia pathogens and an in vivo bacterial challenge study in cobia should be performed to assess the therapeutic efficacy of FF when administered at the optimal dosing regimen.

## 5 Conclusion

FF appears to be an effective option for managing bacterial diseases in cobia, primarily due to its desirable PK characteristics (namely, high serum concentration, large AUC, and moderately long *t*_1/2K_) and relatively short WDT in the muscle/skin (5 days). Antibiotic use in cobia aquaculture warrants greater research attention. Future studies should focus on the drug efficacy profile using an in vivo bacterial challenge to validate the determined optimal dosage. In addition, toxicological studies should also be conducted to evaluate the drug safety profile and determine the maximum tolerable dose. Finally, as environmental factors, especially water temperature, play a crucial role in PK characteristics, further investigation into drug behavior under varying environmental conditions is also necessary.

## Author Contributions

**Jou‐An Shih:** writing – original draft, data curation. **Tirawat Rairat:** writing – original draft, formal analysis, data curation. **Yi‐Ping Lu:** conceptualization, writing – review and editing, resources. **Chi‐Ming Wu:** experiment, data curation. **Niti Chuchird:** writing – review and editing. **Chi‐Chung Chou:** conceptualization, writing – review and editing, supervision, project administration, resources.

## Funding

This work was supported by the Bureau of Animal and Plant Health Inspection Agency, Ministry of Agriculture, Executive Yuan, Taiwan, grant number 113AS‐6.2.1‐BQ‐B1.

## Disclosure

The authors have nothing to report.

## Conflicts of Interest

The authors declare no conflicts of interest.

## Supporting information


**Table S1:** Analytical validation parameters for HPLC‐FLD detection of florfenicol (FF) and florfenicol amine (FFA) in cobia matrices.
**Figure S1:** Representative chromatograms of blank, spiked (5 μg/mL FF/FFA), and incurred samples following extraction. The incurred serum sample is the PK at 8 h (above), and the incurred muscle/skin is the WDT study on Day 1 after final dose (below).
**Figure S2:** The concentration‐time curves for individual animals.

## Data Availability

The data that support the findings of this study are available from the corresponding author upon reasonable request.
