# Supplementary material for: Pharmacokinetics, Optimal Dosages, and Withdrawal Time of Florfenicol in Cobia ( Rachycentron canadum ) After Oral Administration via Medicated Feed
Source: J Vet Pharmacol Ther. 2026 Jan 31;49(3):343–51. doi: 10.1111/jvp.70049 (PMC13159795; doi:10.1111/jvp.70049)
Supplement: Supplementary file 1 — Table S1: Analytical validation parameters for HPLC‐FLD detection of florfenicol (FF) and florfenicol amine (FFA) in cobia matrices. Figure S1: Representative chromatograms of blank, spiked (5 μg/mL FF/FFA), and incurred samples following extraction. The incurred serum sample is the PK at 8 h (above), and the incurred muscle/skin is the WDT study on Day 1 after final dose (below). Figure S2: The concentration‐time curves for individual animals. [file JVP-49-343-s001.docx]

**Supplementary**

**Table S1.** Analytical validation parameters for HPLC-FLD detection of florfenicol (FF) and florfenicol amine (FFA) in cobia matrices.

|  |  | Linear range (µg/mL) | LOD (ng/mL) | LOQ (ng/mL) | Weight r² | Precision (%) | Recovery (%) |
| --- | --- | --- | --- | --- | --- | --- | --- |
| Standard | FF | 0.05–25 | 8 | 26 | 0.9995 | <3% | NA |
|  | FFA | 0.05–25 | 7 | 21 | 0.9996 | <3% | NA |
| Serum | FF | 0.1–25 | 10 | 40 | 0.9987 | <7% | 95–102 |
|  | FFA | 0.1–25 | 10 | 40 | 0.9986 | <7% | 81–90 |
| Skin-on muscle | FF | 0.1–5 | 30 | 80 | 0.9964 | <6% | 86–94 |
|  | FFA | 0.1–5 | 20 | 70 | 0.9967 | <4% | 80–85 |

**
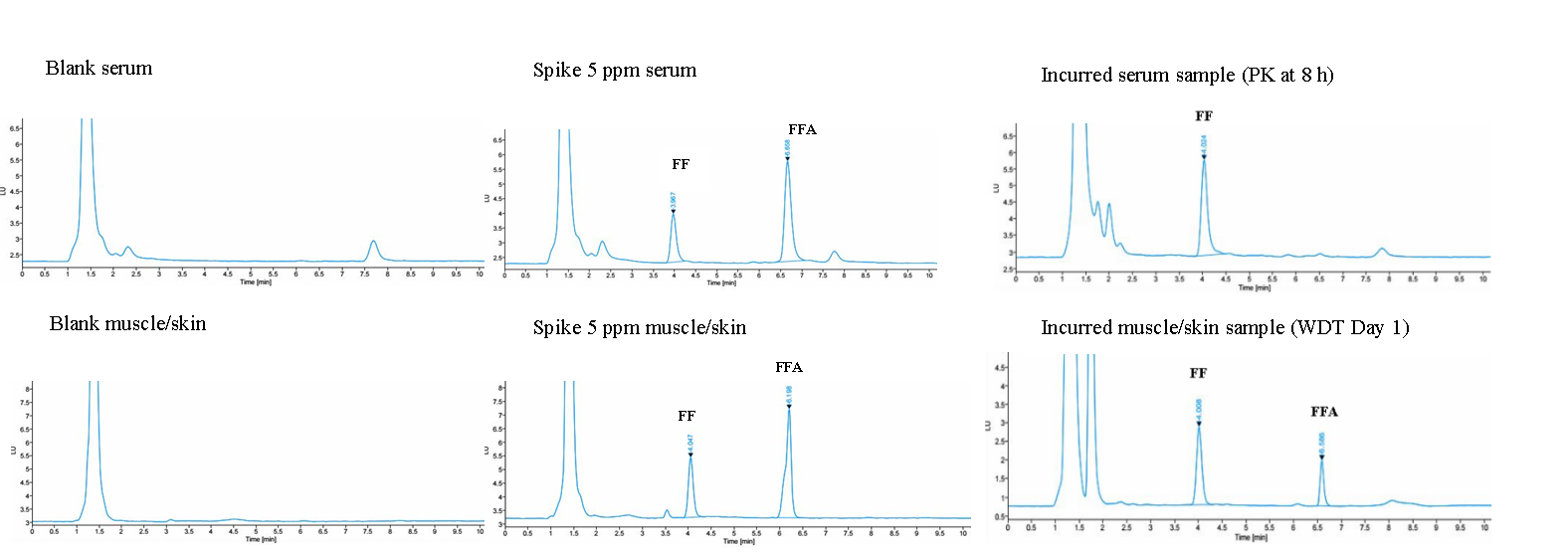
**

**Figure S1.** Representative chromatograms of blank, spiked (5 µg/mL FF/FFA), and incurred samples following extraction. The incurred serum sample is the PK at 8 h (above), and the incurred muscle/skin is the WDT study on Day 1 after final dose (below).


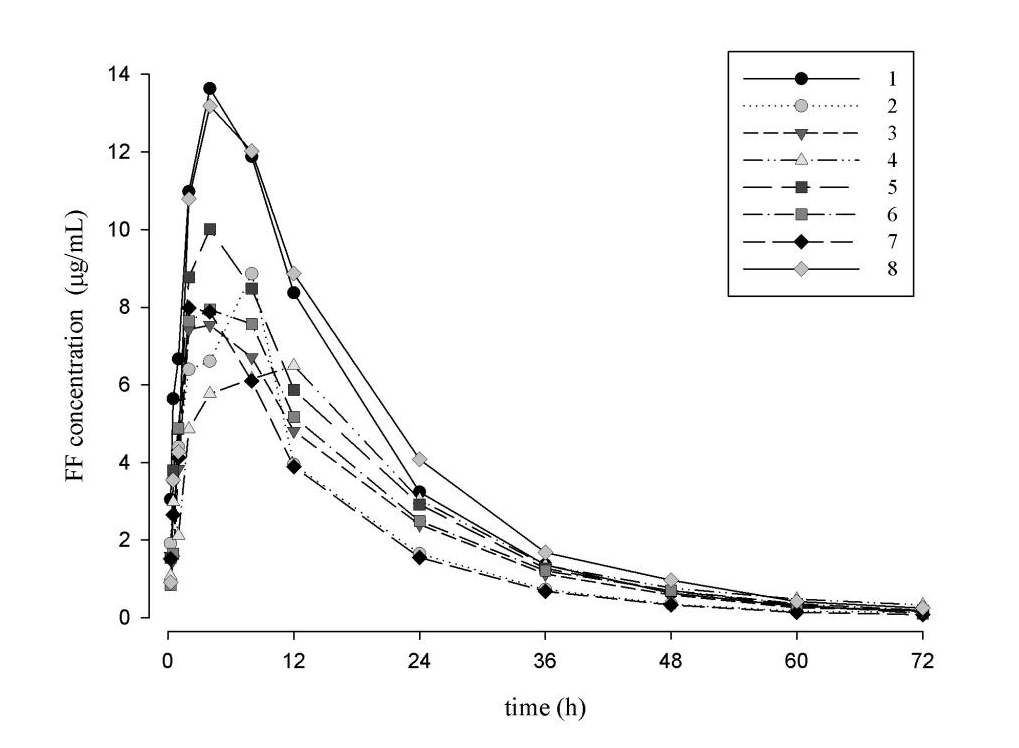


**Figure S2.** The concentration-time curves for individual animals.
